# A New Molecular Phylogeny and a New Genus, *Pendulorchis*, of the *Aerides–Vanda* Alliance (Orchidaceae: Epidendroideae)

**DOI:** 10.1371/journal.pone.0060097

**Published:** 2013-04-05

**Authors:** Guo-Qiang Zhang, Ke-Wei Liu, Li-Jun Chen, Xin-Ju Xiao, Jun-Wen Zhai, Li-Qiang Li, Jing Cai, Yu-Yun Hsiao, Wen-Hui Rao, Jie Huang, Xue-Yong Ma, Shih-Wen Chung, Lai-Qiang Huang, Wen-Chieh Tsai, Zhong-Jian Liu

**Affiliations:** 1 Shenzhen Key Laboratory for Orchid Conservation and Utilization, The National Orchid Conservation Center of China and The Orchid Conservation and Research Center of Shenzhen, Shenzhen, China; 2 The Center for Biotechnology and BioMedicine, Graduate School at Shenzhen, Tsinghua University, Shenzhen, China; 3 South China Botanical Garden, Chinese Academy of Science, Guangzhou, China; 4 Graduate University of Chinese Academy of Sciences, Beijing, China; 5 Department of Life Sciences, National Cheng Kung University, Tainan City, Taiwan; 6 Department of Botany, Taiwan Forestry Research Institute, Taipei, Taiwan; 7 Institute of Tropical Plant Sciences and Orchid Research Center, National Cheng Kung University, Tainan City, Taiwan; 8 College of Forestry, South China Agricultural University, Guangzhou, China; 9 Landscape College of Fujian Agriculture and Forestry University, Fuzhou, China; George Washington University, United States of America

## Abstract

**Background:**

The *Aerides–Vanda* alliance is a complex group in the subtribe Aeridinae (subfamily Epidendroideae, Orchidaceae). Some phylogenetic systems of this alliance have been previously proposed based on molecular and morphological analyses. However, several taxonomic problems within this alliance as well as between it and its allies remain unsolved.

**Methodology/Principal Findings:**

We utilized ITS and five plastid DNA regions in this phylogenetic analysis. Consensus trees strongly indicate that the *Aerides–Vanda* alliance is monophyletic, and the 14 genera of this alliance can be grouped into the following clades with 14 subclades: 1. *Aerides*, comprising two subclades: *Rhynchostylis* and *Aerides*; 2. *Ascocentropsis*; 3. *Papilionanthe*; 4. *Vanda*, comprising five subclades: *Neofinetia*, *Christensonia*, *Seidenfadenia*, *Ascocentrum*, and *Vanda–Trudelia*, in which *Vanda* and *Trudelia* form a subclade; 5. *Tsiorchis*, comprising three subclades: *Chenorchis*, *Tsiorchis*, and two species of *Ascocentrum*; 6. *Paraholcoglossum*; and 7. *Holcoglossum*. Among the 14 genera, only *Ascocentrum* is triphyletic: two species of the *Ascocentrum* subclade, an independent subclade *Ascocentrum* subclade in the *Tsiorchis* clade; the *Ascocentrum* subclade in the *Vanda* clade; and one species in the *Holcoglossum* clade. The *Vanda* and *Trudelia* species belong to the same subclade. The molecular conclusion is consistent with their morphological characteristics.

**Conclusions:**

We elucidate the relationship among the 14 genera of the *Aerides–Vanda* alliance. Our phylogenetic results reveal that the *Aerides–Vanda* alliance is monophyletic, but it can be divided into 14 genera. The data prove that *Ascocentrum* is triphyletic. Plants with elongate-terete leaves and small flowers should be treated as a new genus, *Pendulorchis*. *Saccolabium himalaicum* (*Ascocentrum himalaicum*) should be transferred to *Pendulorchis*. *Ascocentrum pumilum*, endemic to Taiwan, should be transferred to *Holcoglossum*. A new combination, *Holcoglossum pumilum*, was also established. *Trudelia* should not be recognized as an independent genus. Two new species, *Pendulorchis gaoligongensis* and *Holcoglossum singchianum*, were described as well.

## Introduction

Orchidaceae is possibly the largest family of angiosperms with almost 25 000 species. The traditional typological classification divides Orchidaceae into Apostasioideae, Cypripedioideae, Spiranthoideae, Orchidoideae, and Epidendroideae [1]. Recently, Spiranthoideae has been integrated into Orchidoideae, where a new subfamily Vanilloideae was established [2] based on the affinity analysis of internal transcribed spacer (ITS), *trn*L-F, and *mat*K sequences. Epidendroideae is a subfamily that accounts for more than 80% of the orchid species. The *Aerides–Vanda* alliance described in this study is a member of the subtribe Aeridinae and an advanced but complex group in Epidendroideae.

Tsi [3] and Christenson [4]–[7] identifiedthe *Aerides–Vanda* alliance by a comparative analysis of *Holcoglossum* and its allied genera, such as *Vanda*, *Papilionanthe*, *Ascocentrum*, *Aerides*, *Rhynchostylis*, *Seidenfadenia*, *Trudelia* [8], and *Neofinetia*. Subsequently, the following genera were established within the alliance: *Christensonia* [9], *Chenorchis* [10], *Paraholcoglossum* [11], *Tsiorchis* [11], and *Ascocentropsis* [12]. Consequently, 14 genera were included in the alliance. However, the *Aerides–Vanda* alliance is somewhat ambiguous in taxonomic literature, i.e., the same species may be classified under different genera [2], [13]. This confusion arises from using partly overlapping morphological characteristics to distinguish one genus from another. Similarly, Seidenfaden [14] pointed out that “The difficulties arise because we again and again meet with species that can be accommodated in a genus only by widening such a generic circumscription until the situation becomes completely blurred.” Christenson [4] conducted a branch analysis of this alliance (excluding *Neofinetia* and genera subsequently established) using 11 features, and divided the *Aerides–Vanda* alliance into two branches. One branch comprises *Vanda* and *Ascocentrum*, and the other branch includes *Holcoglossum*, *Papilionanthe*, *Aerides*, *Rhynchostylis*, and *Seidenfadenia*. The latter branch is further divided into the three sub-branches, namely, *Aerides*, *Papilionanthe*, and *Holcoglossum–Rhynchostylis–Seidenfadenia* subclades.

In the *Aerides–Vanda* alliance, Garay [15] placed *Papilionanthe* between *Vanda* and *Aerides*, but it is more closely linked to *Aerides*. By contrast, Jin [16] considered *Vanda* as a relatively primal genus of this alliance.

The members of *Ascocentrum* considerably differ in the shapes of their leaves, which can be divided into two types: subterete and nearly lorate leaves. Based on the morphological analysis of the mid-lobe of the lip, stipe, and spur of this genus, Jin [16] stated that *Ascocentrum* maybe grouped between *Aerides* and *Seidenfadenia* because the bilobed uplifted rostellum is unique to *Ascocentrum* and *Seidenfadenia* in the *Aerides–Vanda* alliance, and the leaves of some *Ascocentrum* species are similar to those of *Seidenfadenia*. However, Christenson [4] classified *Ascocentrum* under the same branch where *Vanda* belongs because of its notch-tipped leaves. This finding indicates that the *Ascocentrum* species with terete leaves do not belong to the *Vanda–Ascocentrum* branch.

Christenson [4] inferred that *Holcoglossum* and *Seidenfadenia* are two parallel evolutive branches, whereas Jin [16] considered *Seidenfadenia* to be more evolutive because of the special structure of its spur and rostellum. Most *Seidenfadenia*, *Holcoglossum*, and *Ascocentrum* species have closely similar vegetative organs, specifically, a very short stem and subterete leaves with a ventrally longitudinal groove. Their distribution areas usually overlap with one another, and they have very similar habits. However, the floral structures of these three genera are distinct, particularly their rostellum, pollinia, and stipe. The vegetative comparison in these three genera can be considered as their adaptation to similar habitats [16]. Given that this finding is only a speculation, molecular confirmation is still necessary.

*Rhynchostylis* is relatively close to *Vanda* in terms of its morphological structures, including its robust habit, entirely or slightly trilobed labellum, bilaterally compressed spur with its apex pointing backward, and two cleft pollinia. This species is a relatively primitive genus in the *Aerides–Vanda* alliance. However, its stipe is long and narrows downwards, which make it appear specialized.

Christenson [4] performed the initial branch analysis of the *Aerides–Vanda* alliance and stated that the generic relation of this alliance may have undergone considerable changes after thoroughly researching each genus. The character status of some genera has changed, but some rather ambiguous genera are reclassified. For example, although *Holcoglossum* is polymorphic, it has been treated as a single genus until recently [17]–[19]. However, Liu et al. [11] divided the *Holcoglossum* alliance into three genera, namely, *Holcoglossum*, *Tsiorchis*, and *Paraholcoglossum*, based on further molecular and morphological analyses of more taxa under this alliance and its allied groups. The two new genera were treated by Jin [18] and Fan et al. [19] as either subgeneric or sectional rank.

Although some molecular and morphological systems of this alliance have been proposed in previous studies [2], [20], the relationships among the members of this alliance are unclear. Two species of *Papilionanthe* have been placed in the section *Nujiangensia* of *Holcoglossum* after molecular analysis [11]. Thus, to seek clarification of the *Aerides–Vanda* alliance phylogenetically, molecular and morphological analyses of more species are necessary.

The two recently published genera, *Ascocentropsis* and *Chenorchis*, which genetically belong to the *Aerides–Vanda* alliance, are both monotypic. *Ascocentropsis* has been established based on *Ascocentrum pusillum* [12], whereas *Chenorchis* is perceived to be genetically related to *Holcoglossum* and *Ascocentrum* [10].

In this study, we focused on improving the sampling of the *Aerides–Vanda* alliance to facilitate a more accurate reconstruction of the phylogenetic relationships. We collected specimens of 68 species under the 14 genera of the *Aerides–Vanda* alliance and its three allied genera, with emphasis on *Holcoglossum*, *Paraholcoglossum*, *Tsiorchis*, *Chenorchis*, *Ascocentrum*, *Neofinetia*, *Seidenfadenia*, *Christensonia*, *Trudelia*, and *Ascocentropsis*. Based on the molecular and morphological analyses, we provided a well-supported phylogenetic resolution for the placement of the 14 genera in the *Aerides–Vanda* alliance.

## Results

The DNA sequences of 70 taxa, including 68 species of 17 allied genera and two species of *Cymbidium* as outgroup, were obtained and analyzed. The DNA sequences of all species of *Holcoglossum*, three species of *Paraholcoglossum*, two species of *Tsiorchis*, six species of *Ascocentrum* and one new species similar to *Ascocentrum himalaicum*, one species of *Chenorchis*, three species of *Neofinetia*, six species of *Vanda*, two species of *Papilionanthe*, and three species of *Aerides* were newly obtained, and other species were accessed from GenBank. Tables 1 and 2 provide the detailed sequence information, aligned length, numbers of variable sites, parsimony informative sites, tree statistics for maximum parsimony (MP) analysis, and the best-fit model selected by Modeltest.

**Table 1:** Statistics from the analysis datasets.

Information	No. of taxa	Aligned length	No. variablecharacters	No. informativecharacters (%)	Treelength	Consistencyindex	Retentionindex
ITS	67	736	379	236(32.1%)	991	0.5550	0.7442
*mat*K	68	1640	359	186(11.3%)	589	0.7029	0.7528
*trn*L-F	62	1541	395	221(14.3%)	643	0.7372	0.8126
*psb*A*-trn*H	42	1025	106	53(5.2%)	127	0.8740	0.8621
*atp*I*-atp*H	39	886	223	176(19.9%)	268	0.9067	0.9351
*trn*S*-trnf*M	39	1122	110	65(5.8%)	134	0.8881	0.9198
cpDNA	68	6214	1193	701(11.3%)	1885	0.7220	0.7720
Combined	70	6950	1572	937(13.5%)	2965	0.6445	0.7379

**Table 2:** Best-fit model and parameter for the analysis datasets.

Region	AIC selectmodel	Base frequencies				substitution model(rate matrix)						I	G
		A	C	G	T	A-C	A-G	A-T	C-G	C-T	G-T		
ITS	GTR+I+G	0.1717	0.3255	0.3616	0.1411	1.0095	3.4540	0.9949	0.5939	4.4265	1.0000	0.2185	0.9418
*mat*K	GTR+I+G	0.3229	0.1532	0.1424	0.3814	1.0576	1.2520	0.1481	0.4903	1.0083	1.0000	0.4153	0.9585
*trn*L-F	TVM+I+G	0.3683	0.1222	0.1292	0.3802	1.2574	1.1553	0.7785	0.1362	1.1553	1.0000	0.3645	0.3229
*psb*A*-trn*H	GTR+I+G	0.3282	0.1548	0.1606	0.3563	1.0275	0.9166	0.8045	0.1412	0.2959	1.0000	0.7543	0.7690
*atp*I*-atp*H	K81uf+G	0.3534	0.1267	0.1812	0.3387	1.0000	1.0952	0.2983	0.2983	1.0952	1.0000	0.0000	1.4207
*trn*S*-trnf*M	K81uf+G	0.3409	0.1666	0.1457	0.3469	1.0000	1.6369	0.4514	0.4514	1.6369	1.0000	0.0000	0.2414
cpDNA	GTR+I+G	0.3388	0.1508	0.1518	0.3587	0.9560	1.1765	0.5484	0.2459	0.9456	1.0000	0.5416	0.7061
Combined	TIM+I+G	0.3157	0.1757	0.1786	0.3301	1.0000	1.7855	0.5976	0.5976	1.5632	1.0000	0.4865	0.5983

### ITS Analysis

A total of 67 taxa were analyzed. Most genera form independent clades or subclades. The generic relationships are mostly well resolved. However, the phylogenetic positions of some species are unclear, such as those of *Paraholcoglossum auriculatum*, *Seidenfadenia mitrata*, *Rhynchostylis gigantean*, *Rhynchostylis retusa*, and *Aerides odorata*. Figs. S1, S2, and S3 provide the detailed results.

### Chloroplast DNA Analysis

In this analysis, chloroplast DNA (cpDNA), including *trn*L-F, *mat*K, *psb*A*-trn*H, *atp*I*-atp*H, and *trn*S*-trnf*M regions were combined as a dataset for analysis. A total of 68 taxa were analyzed. Most genera form independent clades or subclades. The generic relationships are mostly well resolved. However, the position of *Papilionanthe hookeriana* and the phylogenetic relationships of *Ascocentrum*, *Christensonia* and *Seidenfadenia mitrata* are unclear. Figs. S4. S5, and S6 provide the detailed results.

### Combined Dataset Analysis

**Figure 1:**
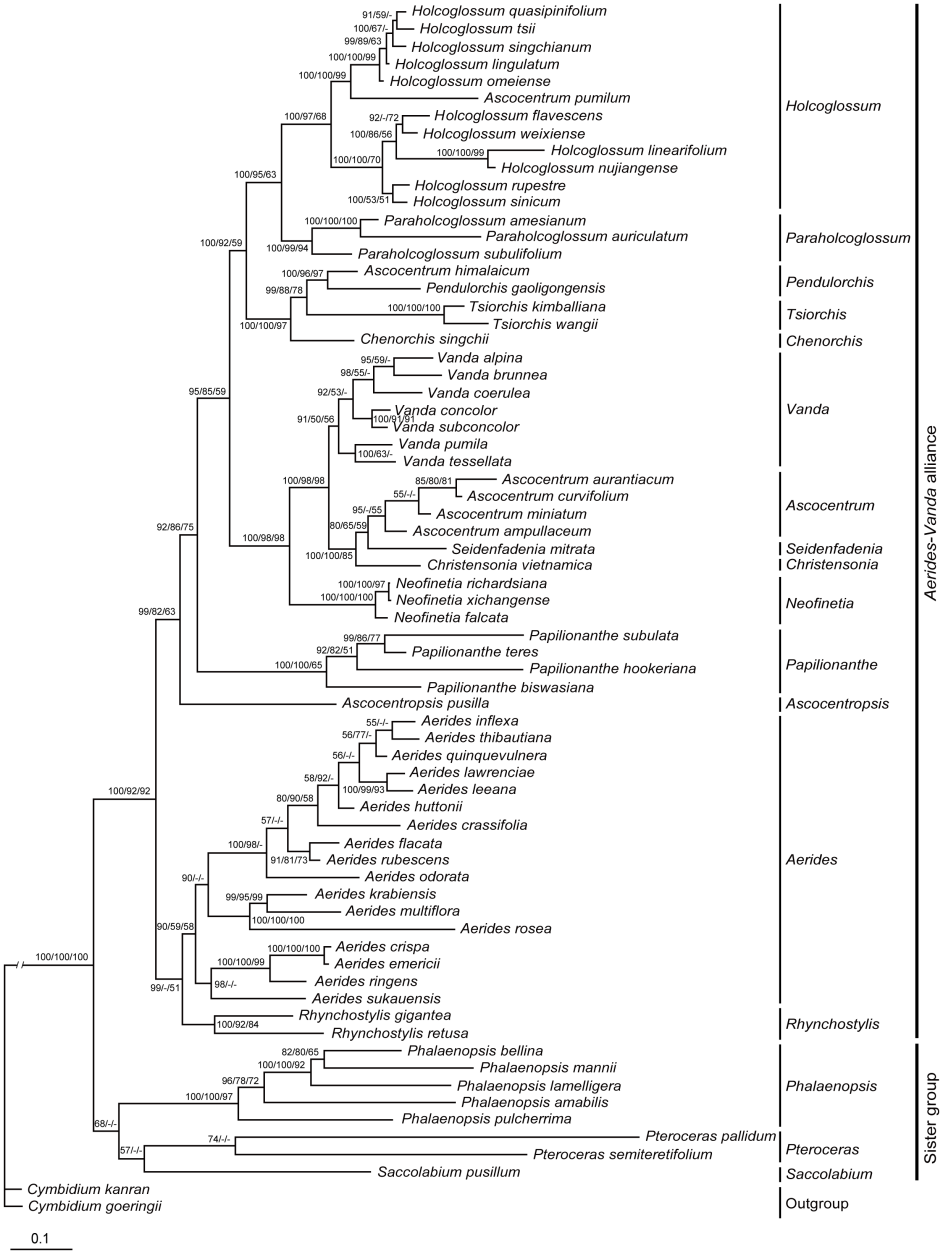
Phylogram obtained from Bayesian inference analysis of the combined nrDNA ITS and cpDNA data. Numbers near the nodes are Bayesian posterior probabilities (×100) and bootstrap percentages (PP left, BB_ML_ middle, BB_MP_ right), respectively. “–” indicates that the node was not supported in ML and MP analysis.

We also combined all datasets into a single dataset for the phylogenetic analysis of the *Aerides–Vanda* alliance. The strict consensus trees strongly suggest that the *Aerides–Vanda* alliance is monophyletic, and the 14 genera under this alliance can be divided into the following 7 clades with 14 subclades: 1. *Aerides*, comprising two subclades: *Rhynchostylis* and *Aerides*; 2. *Ascocentropsis*; 3. *Papilionanthe*; 4. *Vanda*, comprising five subclades: *Neofinetia*, *Christensonia*, *Seidenfadenia*, *Ascocentrum*, and *Vanda*; 5. *Tsiorchis*, comprising three subclades: *Chenorchis*, *Tsiorchis*, as well as one species of *Ascocentrum* and one new species, *Pendulorchis gaoligongensis*; 6. *Paraholcoglossum*; and 7. *Holcoglossum*. Among these clades, only *Ascocentrum* is triphyletic and comprises two subclades. One is the *Ascocentrum* subclade that is related to the *Seidenfadenia* subclade; the other is the *Ascocentrum himalaicum* and *Pendulorchis gaoligongensis* form an independent subclade that is much more closely related to *Tsiorchis* than to *Ascocentrum* subclade. Another species, *Ascocentrum pumilum*, should be transferred to the *Holcoglossum* clade. *Trudelia* species do not form an independent subclade but belong to the *Vanda* subclade. The molecular conclusion is consistent with their morphological characteristics. Fig. 1 provide the detailed results.

## Discussion

### Data Analysis

In this study, six DNA regions were utilized, including one nuclear (ITS) and five plastid (*trn*L-F, *mat*K, *psb*A*-trn*H, *atp*I*-atp*H, and *trn*S*-trnf*M) regions. The results show that most phylogenetic relationships based on ITS agree with the plastid datasets and their combination, but some genera such as *Rhynchostylis*, *Siedenfadenia* and *Paraholcoglossum* appear to have some different phylogenetic relationships between ITS and plastid data. Such differences may result from intergeneric hybridization or introgression at some point during the evolution of these genera, which should need further study to testify. We performed an incongruence length difference test between ITS with cpDNA, and the result shows incongruence to a certain extent between the ITS and plastid regions (*P* = 0.01), but it did not affect the whole phylogenetic relationship. In fact, different genes (including ITS and cpDNA) are incongruent in many cases. Different genes are known to have different evolutionary rates and can provide different evolutionary information. Thus, we need to use more than one gene to assess their phylogenetic relationship because of their incongruence. Based on our experience and those of other researchers, the obtained phylogenetic relationship is better when more genes are used. The combination dataset still produced the best trees. Among them (Fig. 1), most species belong to their phylogenetic clades or subclades and most nodes have good support. Therefore, we believe that combining a nuclear ITS and plastid regions to solve the phylogenetic relationship is appropriate.

### Overall Tree and Effect of Taxon Sampling

**Figure 2:**
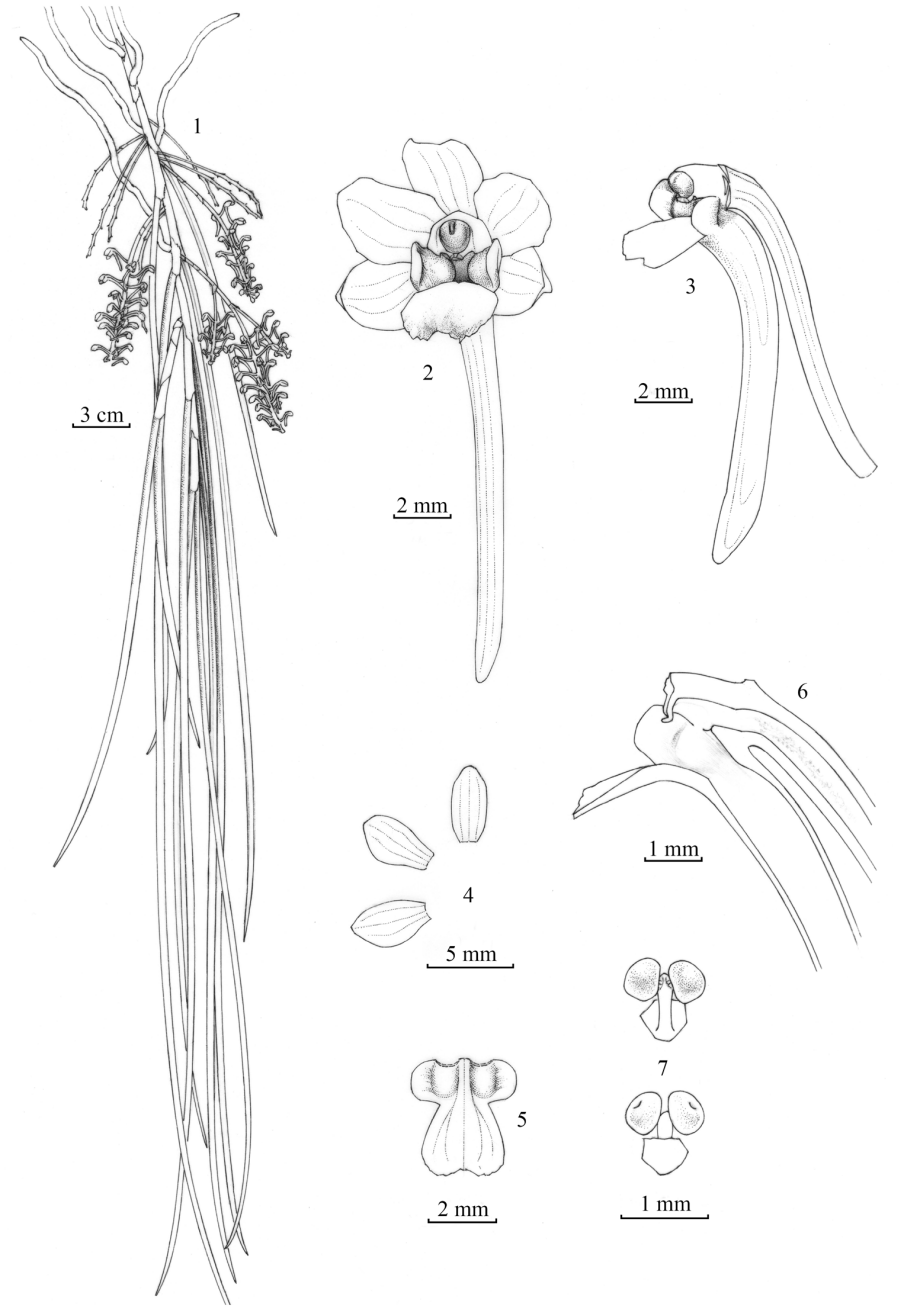
*Pendulorchis gaoligongensis* G. Q. Zhang, Ke Wei Liu et Z. J. Liu. 1.Flowering plant. 2.Flower, front view. 3. Column and lip, side view. 4. Dorsal sepal, petal and lateral sepal. 5. Lip. 6. Column and lip, longitudinal section. 7. Pollinarium, front view and back view. Drawn by X. Y. Ma from the type Z. J. Liu 5871 (NOCC).

The phylogenetic analyses identified the following seven major clades: 1. *Aerides*, comprising two subclades: *Rhynchostylis* and *Aerides*; 2. *Ascocentropsis*, monotypic; 3. *Papilionanthe*, comprising four species; 4. *Vanda*, comprising five subclades, *Neofinetia*, *Christensonia*, *Seidenfadenia*, four species of *Ascocentrum*, and *Vanda*; 5. *Tsiorchis*, comprising three subclades: *Chenorchis*, *Tsiorchis*, as well as two species of *Ascocentrum* and *Pendulorchis gaoligongensis* (PP 1.00, BS_ML_ 100 and BS_MP_ 97); 6. *Paraholcoglossum*; and 7. *Holcoglossum*, comprising all species of *Holcoglossum* and one species of *Ascocentrum* (PP 1.00, BS_ML_ 95 and BS_MP_ 63). Among the 14 genera, only *Ascocentrum* is triphyletic and comprises three subclades. First is the *Ascocentrum* subclade that is related to the *Seidenfadenia* subclade; second is the *Ascocentrum himalaicum* and *Pendulorchis gaoligongensis* form an independent subclade that is much more closely related to *Tsiorchis* than to *Ascocentrum*; and third is a species, *Ascocentrum pumilum*, that should be transferred to the *Holcoglossum* clade. The parsimony, maximum likelihood, and Bayesian approaches for the combined dataset result in similar tree topologies, with the identification of seven well-supported major clades (Fig. 2). This topology partially agrees with the proposal of Liu et al. [11].

The seven clades mostly receive moderate to high support (over 50). Species of the *Holcoglossum* clade are related to the species of the *Paraholcoglossum* clade (PP 1.00, BS_ML_ 97 and BS_MP_ 68). *Ascocentrum himalaicum*, often placed in *Holcoglossum*, is related to the *Tsiorchis* subclade (PP 0.99, BS_ML_ 88 and BS_MP_ 78). The *Ascocentrum* species are divided into three groups, belonging to the *Tsiorchis*, *Vanda*, and *Holcoglossum* clades respectively.

### Morphological Characteristics and Distribution Analyses

The morphological characteristics of the *Aerides–Vanda* alliance support its division into the following 14 genera. (1) *Rhynchostylis*, which includes two species that are both large plants with flat leaves, laterally compressed and backward-pointing spur, cleft pollinia with narrow stipe much longer than the pollinia, and a small viscidium. This genus is widespread in tropical Asia north to south China. (2) *Aerides* includes 17 species with flat leaves, elongated column, appendiculate spur, bifid rostellum, cleft pollinia, and semicircular viscidium. This genus is widespread in tropical Asia from north China to the Himalayas. (3) *Ascocentropsis* is a monotypic genus similar to *Ascocentrum*, from which it differs by having more or less cross-shaped pollinarium with sulcate or split pollinia, visible caudicle, narrowly linear stipe much longer than either the pollinia or viscidium, and strongly incurved side-lobes of the lip. This genus is found in Thailand. (4) *Papilionanthe* includes four species with terete leaves, large flowers, trilobed lip attached to the column foot, elongated rostellum, and cleft pollinia attached by a broadly triangular or subquadrate stipe to a large viscidium. This genus is distributed in China, India, and Southeast Asia. (5) *Neofinetia* has flat narrow leaves, cleft pollinia, footless column, and narrow stipe much longer than pollinia. This genus is found in China, Japan, and Korea. (6) *Christensonia* is a monotypic genus that exhibits a mosaic of characters found in its closely related genera *Aerides* and *Rhynchostylis.* This genus, distributed in Vietnam, differs from the former by having a footless column and from the latter by a clearly three-lobed lip [9]. (7) *Seidenfadenia*, a monotypic genus similar to *Holcoglossum*, from which it differs by having a pouched and backward-pointing spur, is found in Thailand and Myanmar. (8) *Ascocentrum miniatum*, *Ascocentrum ampullaceum*, *Ascocentrum curvifolium*, and *Ascocentrum aurantiacum* have flat leaves, similar sepals and petals, lip firmly adnate to the column base with suberect lateral lobes adnate to column, and oblong mid-lobe thickening at the base. These species are found in Southeast Asia and the Himalayas. (9) *Vanda* includes five species of *Vanda* and two species transferred back from *Trudelia* (*T. alpina* and *T. pumila*), with cleft pollinia, footless column, and a broad stipe shorter than pollinia. This genus is widespread in tropical Asia extending to New Guinea and Australia. (10) *Chenorchis* is a monotypic genus with clavate rachis, three-lobed lip with its side-lobes from both lower sides of the mid-lobe, and large rostellum conspicuously broader than the column. This species is distributed in China (West Yunnan). (11) *Tsiorchis*, consisting of *T. kimballiana* and *T. wangii*, is characterized by their long-cylindrical spur, cleft pollinia, distinct caudicle attached to a common stipe, and purple-marked lip. This genus is found in China (Guangxi, Yunnan), Laos, Myanmar, Thailand, and North Vietnam. (12) *Ascocentrum himalaicum* and new species (*Pendulorchis gaoligongensis*), pendulous plant with terete leaves 30 cm to 60 cm long, very small flowers, white lip with very long spur, and very short sepals and petals. These plants are distributed in China (Yunnan), Bhutan, NE India, and Myanmar. (13) *Paraholcoglossum*, consisting of *P. amesianum*, *P. subulifolium*, and *P. auriculatum*, is characterized by a lip shallowly saccate at the base, a ridged callus at the sac entrance, a mid-lobe clawed at the base, and oblong stipe. These plants are found in China (Yunnan, Hainan), India, Laos, Myanmar, Thailand, and Vietnam. (14) *Holcoglossum*, which has 13 species including one new species and one new combination, is characterized by horn-shaped spur, porate pollinia directly attached to a common stipe, and white lip. This genus is found in China (Taiwan, Fujian, Sichuan, Guangxi, and Yunnan) and North Vietnam.

### *Holcoglossum* Clade

This clade comprises all species of *Holcoglossum*, including one species transferred from *Ascocentrum*. Our data strongly suggest (PP1.00) that the *Holcoglossum* clade is related to the *Paraholcoglossum* clade. However, *Tsiorchis* should be placed distantly outside the *Holcoglossum* clade. *Papilionanthe* is treated as an independent clade outside the *Holcoglossum* clade. The aforementioned four genera have marked differences in morphology (Fig. 1). The phylogenetic relationships between *Holcoglossum* and its allied genera are well resolved, and all data sets supported the recognition of *Papilionanthe*. The *Holcoglossum* alliance is divided into the *Tsiorchis*, *Paraholcoglossum*, and *Holcoglossum* clades (PP1.00).

*Holcoglossum* is treated as a genus of 13 species, including a new species, *H. singchianum*, and a new combination. They all combine to form a homologous subclade. The new species is related to *Holcoglossum quasipinifolium*, but distinguishable from it by some marked characteristics and should be treated as a new species.

### *Paraholcoglossum* Clade

From the tree of the combined dataset, the *Paraholcoglossum* clade is related to the *Holcoglossum* clade. *Paraholcoglossum* comprises three species: *P. subulifolium*, *P. auriculatum*, and *P. amesianum*. Morphologically, this genus differs from *Holcoglossum* by a lip that is saccate (not spurred) at the base, a ridged (not crested or fleshy) callus at the sac entrance (not mid-lobe base), and an oblong (not tapering) stipe [11].

Three distinct pollination systems are observed in the *Holcoglossum* and *Paraholcoglossum* alliance, specifically, the autogamy in *Paraholcoglossum amesianum* [21], beetle pollination in *Holcoglossum rupestre* [22], and bee pollination in *Holcoglossum nujiangense* [23].

### *Rhynchostylis–Aerides* Clade

This clade consists of the two subclades *Rhynchostylis* and *Aerides*. *Rhynchostylis* has three or four species, and its two Chinese species, which combine to form a subclade, were analyzed in this study. This study further suggests that *Rhynchostylis* is an independent genus. *Aerides* has approximately 20 species, among which 16 species were analyzed in this study. They combine to form a basal subclade of the *Aerides–Vanda* alliance and another basal subclade related to the *Rhynchostylis* subclade. The topology of *Aerides* partially agrees with that described by Kocyan et al. [2].

### *Papilionanthe* Clade

This clade is composed of all 12 *Papilionanthe* species. The four species selected in this study combine to form a group distantly related to *Holcoglossum*, which contradicts previous findings [11]. *Papilionanthe* has an elongated stem, terete leaves, two- or three-lobed mid-lobe of the lip, cylindrical or horn-shaped spur, and short cleft pollinia. These characteristics relatively leave an evolutionary trace in *Tsiorchis, Paraholcoglossum*, and *Holcoglossum*.

### *Ascocentropsis* Clade

The *Ascocentropsis* clade includes a monotypic genus formerly established based on *Ascocentropsis pumila* [12]. The molecular data from this study strongly support the independent position of this clade at the generic level, and prove that the *Ascocentropsis* clade is a basal clade compared with the *Holcoglossum* clade.

### *Vanda (Trudelia)–Ascocentrum–Neofinetia* Clade

This group is a multifarious clade composed of the following subclades: (1) *Neofinetia* comprising the monophyletic genus *Neofinetia* with three species; (2) *Christensonia*, a monotypic genus that is considered independent at the generic level and related to the *Seidenfadenia* and *Ascocentrum* subclades; (3) *Seidenfadenia*, also a monotypic genus and a subclade related to the *Ascocentrum* subclade; (4) *Ascocentrum*, comprising four species with big flowers and flat leaves, i.e., a subclade related to the *Seidenfadenia* subclade. One other species and one new species (*Pendulorchis gaoligongensis*) with small flowers and terete leaves are considered independent genera placed in the *Pendulorchis–Tsiorchis–Chenorchis* clade. *Pendulorchis gaoligongensis* has unique characteristics and should be treated as a new species. *Ascocentrum pumilum* should be transferred to *Holcoglossum*; and (5) *Vanda*, comprising approximately 40 species, seven of which were selected in this study and combined to form a subclade. *Trudelia alpina* and *Trudelia pumila* should be transferred back to *Vanda*.

### *Pendulorchis–Tsiorchis–Chenorchis* Clade

This clade is composed of the following subclades. (1) *Chenorchis* comprising a monotypic genus described in 2008 in China [10]. This species was previously believed [13] to be identical with the Indian plant *Penkimia nagalandensis* [24]. However, further molecular comparative analysis between these two species is needed. The sample in our study is from the type specimen of *Chenorchis*, which provides evidence that it is genetically related to *Tsiorchis* (original *Holcoglossum*) and *Pendulorchis* (original *Ascocentrum*). Thus, these subclades are all related. (2) *Tsiorchis* comprises a genus of two species and originally belongs to *Holcoglossum. Tsiorchis* is related to the *Pendulorchis* subclade, but it is quite distinct from *Holcoglossum* and *Paraholcoglossum* due to its cleft, not its porate. The pollinia each have a distinct caudicle attached to a common stipe and lip marked with purple or dark purple on the mid-lobe and side-lobes. (3) *Pendulorchis* subclade includes a genus of one species that originally belongs to *Ascocentrum* and one new species. These two species are pendulous and have tiny, not fully open flowers. *Pendulorchis* is closely related to *Tsiorchis* and to a lesser extent to *Chenorchis.* These species form the *Pendulorchis–Tsiorchis–Chenorchis* clade.

Our molecular analysis and morphological observation strongly support the recognition of the following as independent genera: *Holcoglossum*, *Paraholcoglossum*, *Tsiorchis*, *Chenorchis*, *Christensonia*, *Seidenfadenia*, *Ascocentropsis*, *Neofinetia*, *Ascocentrum*, *Pendulorchis*, *Papilionanthe*, *Aerides*, *Rhynchostylis*, and *Vanda*. However, the branch analysis by Christenson [4] is only partially supported. We do not support the placement of *Papilionanthe* between *Vanda* and *Aerides*, as previously treated by Garay [15]. Instead, we propose that *Papilionanthe* should be placed between the *Aerides–Rhynchostylis* and *Vanda–Ascocentrum–Neofinetia* alliances. Although this study covered all types of species, not all species of the genera in the *Aerides–Vanda* alliance were considered. Thus, further research is needed to confirm whether all the species in these genera are homologous.

### Conclusion

The *Aerides–Vanda* alliance is confirmed to be monophyletic, but it can be divided into 14 genera, including the recently established *Chenorchis*, *Ascocentropsis*, *Christensonia*, *Seidenfadenia*, *Paraholcoglossum*, and *Tsiorchis*. We elucidate the relationship among the 14 genera of the *Aerides–Vanda* alliance, which comprises 7 main clades with 14 subclades. Its basal groups are *Rhynchostylis* and *Aerides*. Our molecular data prove that *Ascocentrum* is triphyletic. *Ascocentrum pumilum* is closely related genetically to *Holcoglossum* and should be transferred to this genus. One species of *Ascocentrum* and one new species with terete leaves and tiny flowers are treated in this study as a new genus, *Pendulorchis*, which is more closely related to *Tsiorchis* than to *Ascocentrum.* This study suggests that *Vanda alpina* and *Vanda pumila* should not be separated from *Vanda* to form an independent genus, *Trudelia.* A new genus, *Pendulorchis*, and two new species, *Holcoglossum singchianum*, are also described. Three new combinations, *Pendulorchis gaoligongensis*, *Pendulorchis himalaica*, and *Holcoglossum pumilum*, are established as well.

### Taxonomic Treatment

#### *Pendulorchis*

Z. J. Liu, Ke Wei Liu et G. Q. Zhang, gen. nov. [urn:lsid:ipni.org:names: 77125660-1].

#### Diagnosis

Genus novum *Ascocentro* Schlechter ex J. J. Smith et *Tsiorchide* Z. J. Liu, S. C. Chen et L. J. Chen simile, a quibus plantis pendulis, caulibus saepe 13 cm to 24 cm longis foliis 6 to 14 praeditis, inflorescentiis multis (saepe 6 to 15), floribus 17 ad 39, viscidio diametro pollinium fere aequanti bene differt.

#### Description

Epiphytic pendulous plants, with many long and flattened roots. Stem often 13 cm to 24 cm long, enclosed by leaf sheaths, often branched. Leaves 6 to 14, fleshy, deep green, subterete, 30 cm to 60 cm long, 3 mm to 5 mm in diameter, channeled adaxially, jointed and sheathed at the base. Inflorescences often 6 to 15, paniculate or racemose, arising from the axils of the lower leaves, with 17 to 39 flowers; flowers 4 mm to 5 mm in diameter, not fully open; sepals, petals, and lip reddish; dorsal sepal oblong, abaxially carinate; lateral sepals elliptic; petals obovate-elliptic; lip 3-lobed; side-lobe erect, oblong, toward abaxial base strongly concave forming a callus-like structure; mid-lobe spreading forward, obovate, adaxially with three longitudinal midveins; spur cylindric; column stout and short; pollinia two globose, cleft, attached by a common stipe to a large suborbicular viscidium.

#### Type

*Pendulorchis gaoligongensis* G. Q. Zhang, Ke Wei Liu et Z. J. Liu.

#### *Pendulorchis gaoligongensis*

G. Q. Zhang, Ke Wei Liu et Z. J. Liu, sp. nov. Fig. 2, Fig. S7. [urn:lsid:ipni.org:names: 77125661-1].

#### Type

China, Yunnan, Gaoligongshan, Lushui, 2010 m, growing on the branch of a big tree, 2011. 10. 10, Z. J. Liu 5871 (NOCC).

#### Diagnosis

Species nova *Ascocentro himalaico* (Deb, Sengupta & Malick) Christenson similis, a quo caulibus 14–25 cm longis foliis 6–16 praeditis, inflorescentiis 5–18 floribus 18 ad 41 praebentibus, labello rubello coloro sepala petalasque aequanti, viscidio diametro pollinium fere aequanti bene differt.

#### Description

Epiphytic plants, pendent, with many flattened roots. Stem 14–25 cm long, 4–5 mm in diameter, enclosed by leaf sheaths, often branched. Leaves 6–16, fleshy, deep green, subterete, 40–60 cm long, 4–5 mm in diameter, channeled adaxially, acute at apex, jointed and sheathing at base; sheaths 4–5 cm long. Inflorescences 5–16, racemose, arising from the axils of the lower leaves, 7–15 cm long, with 18–41 flowers; floral bracts broadly ovate, 2–3 mm long; flowers 8–9 mm in diameter, reddish; pedicel and ovary 1.2–1.5 cm long; dorsal sepal oblong, 4–5 mm long, 1.8–2.2 mm wide, rounded at apex; lateral sepal elliptic, 4–5 mm long, 2.5–2.8 mm wide, obtuse at apex; petals obovate-elliptic, 4–5 mm long, 2.2–2.4 mm wide, obtuse at apex; lip 3-lobed; side-lobe erect, oblong, 2–2.5 mm long, 1–1.2 mm wide, obtuse, toward abaxial base strongly concave forming a callus-like structure; mid-lobe spreading forward, obovate, 2.5–3 mm long, 2.5–3 mm wide, adaxially with 3 longitudinal midveins; spur cylindric, 1.2–1.5 cm long, 1.2–1.5 mm thick, obtuse-tipped; column stout and short, 1.8–2 mm long; anther cap purple; pollinia 2, globose, cleft, attached by a common stipe to a large suborbicular viscidium.

#### Flowering period

October–November.

#### Distribution

China, SW Yunnan (Lushui County).

#### Habitat

Epiphytic, on branches of tall trees in evergreen forest at an altitude of 1800–2100 m.

#### *Pendulorchis himalaica*

(Deb, Sengupta & Malick) Z. J. Liu, Ke Wei Liu et X. J. Xiao, comb. nov. [urn:lsid:ipni.org:names: 77125662-1].

#### Basionym

*Saccolabium himalaicum* Deb, Sengupta, & Malick, Bull. Bot. Soc. Bergal 22 (2): 213. 1968.

#### Synonym

*Holcoglossum himalaicum* (Deb, Sengupta, & Malick) Averyanov in Bot. J. (Leningrad) 73 (1) 101–107, 1988; *H. junceum* Z. H. Tsi in Acta Phytotax. Sin. 20 (4): 442. Fig. 1. 1982; *Ascocentrum himalaicum* (Deb, Sengupta, & Malick) Christenson in Notes Bot. Gard. Edinb. 44∶256. 1987.

#### Distribution

China, SW and W Yunnan; Bhutan, NE India, and Myanmar.

#### *Holcoglossum singchianum*

**Figure 3:**
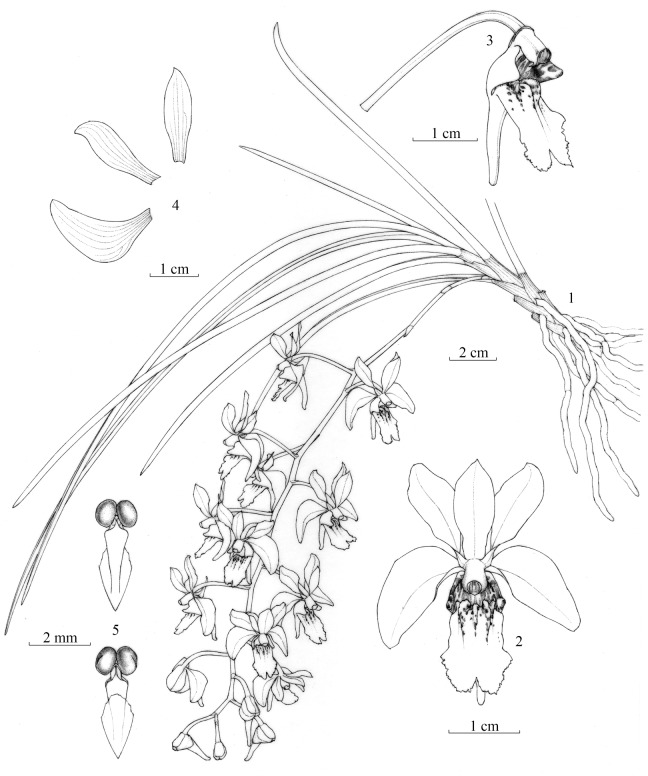
*Holcoglossum singchianum* G. Q. Zhang, L. J. Chen et Z. J. Liu. 1. Flowering plant; 2. Flower, front view; 3. Lip and column, side view; 4. Dorsal sepal, petal, and lateral sepal; 5. Pollinarium, front view and back view.

G. Q. Zhang, L. J. Chen, & Z. J. Liu sp. nov. Fig. 3, Fig. S8. [urn:lsid:ipni.org:names: 77125663-1].

#### Type

Yunnan, Xichou, in forest, on tree trunk, alt. 1300 m. 2009. 04. 30. Z. J. Liu 4532 (NOCC).

#### Diagnosis

Species nova *Holcoglosso linearifolio* similis, a quo differt foliis 3–4 mm in diam., inflorescentia 12- ad 16-flora, lobo intermedio labelli subobovato-rhambico, ejus lobis lateralibus flavis et bruneo-maculatis.

#### Description

Epiphytic plant. Stem nearly ascending, 5–6 cm long, enclosed in persistent leaf sheaths, 7- to 8-leaved. Leaves fleshy, cylindric, 19–37 cm long, 3–4 mm thick, adaxially channeled, acuminate at apex, base dilated into amplexicaul sheaths. Inflorescence 12- to 16-flowered; peduncle 8–9 cm long, with 2 to 3 tubular sheaths; rachis 14–20 cm long; floral bracts broadly ovate, 3–4.5 cm long, obtuse at apex; pedicel and ovary 2.4–3.5 cm long; flowers 3–3.8 cm across; sepals and petals white with a purple midvein; mid-lobe of lip white, purple-spotted on lamellae; side-lobes yellow, brown-spotted; dorsal sepal obovate-elliptic, 1.6–2 cm long, 6–7 mm wide, acuminate at apex; lateral sepals falcate-oblong, 1.7–2.2 cm long, 8–10 mm wide, obtuse at apex; petals obovate-oblong, 1.6–2.1 cm long, 5.5–6.5 mm wide, acuminate at apex; lip 3-lobed; side-lobes erect, apex deeply emarginate forming front and rear lobules; front lobule subovate triangular, 3–3.5 mm long, obtuse at apex; rear lobule subovate; mid-lobe subovate-rhombic, 1.7–2.3 cm long, 1.1–1.3 cm wide, apex fork-shaped, front margin undulate and toothed; spur horn-shaped, 1.7–2.1 cm long, apex slightly obtuse; column 5–6 mm long; pollinia 2, globose. Capsule ellipsoid, ca. 4.5 cm long, 7.5 mm thick.

The new species is similar to *H. linearifolium*, differing by its nearly ascending stem, leaves 3–4 mm thick, inflorescence 12- to 16-flowered, lip with subobovate-rhombic mid-lobe, and yellow side-lobes spotted with brown.

#### Flowering period

November to December.

#### Distribution

China, SE Yunnan (Malipo County).

#### Habitat

Epiphytic, on tree trunks in broad-leaved forests, 1300–1500 m.

#### *Holcoglossum pumilum*

(Hayata) L. J. Chen, X. J. Xiao et G. Q. Zhang comb. nov. [urn:lsid:ipni.org:names: 77125664-1].

#### Basionym

*Saccolabium pumilum* Hayata in Bot. Mag. (Tokyo) 20∶77, 1906.

#### Synonym

*Ascocentrum pumilum* (Hayata) Schlechter in Repert. Spec. Nov. Regni Veg. Beih. 4∶285. 1919; *Ascolabium pumilum* (Hayata) S. S. Ying in Col. Ill. Inding. Orch. Taiwan 1∶54, 1977.

#### Distribution

Taiwan, China.

## Materials and Methods

### Materials

A total of 70 species of 18 genera were analyzed, including all genera of the *Aerides–Vanda* alliance proposed by Tsi [3] and Christenson [4]. These genera include *Holcoglossum*, *Vanda*, *Trudelia*, *Papilionanthe*, *Ascocentrum*, *Aerides*, *Rhynchostylis*, *Seidenfadenia*, *Neofinetia*, and the recently established genera, *Chenorchis*, *Paraholcoglossum*, *Tsiorchis*, *Ascocentropsis*, and *Christensonia*. The analyzed samples comprise the type species of all genera. Two *Cymbidium* species, *C. kanran* and *C. goeringii*, were selected as outgroup [20], [25]. To compare the genera of the *Aerides–Vanda* alliance, we added three relevant genera, *Phalaenopsis*, *Pteroceras*, and *Saccolabium* to the analyzed samples. Table S1 provides detailed information regarding the assessment.

### Amplification and Sequencing

Total DNA was extracted from fresh material or silica gel-dried plant tissue using a Multisource Genomic DNA Miniprep Kit (Axygen Biosciences) following the manufacturer’s instructions. The amplification reaction included total DNA, primers, Ex-Taq buffer, and Ex-Taq DNA polymerase (Takara Bio). The polymerase chain reaction (PCR) profile consisted of an initial 5 min pre-melting stage at 95°C, followed by 30 cycles of 30 s at 95°C (denaturation), 30 s at 50°C to 55°C (annealing temperature was determined based on the primer requirement), and 1 min to 3 min at 72°C (extension time was determined based on the length of the target DNA region), and a final 10 min extension at 72°C.

The amplification of the ITS region was performed using the primer pairs ITS A and ITS B [26]. The *trn*L-F region was amplified with primers c and f [27] or the two sets of primers developed by Liu et al. [11]. For *mat*K sequences, amplification was performed using the primer pair *mat*K-19F and *trn*K-2R [26], and several fragments were amplified using the three sets of primers developed by Liu et al. [11]. The *psb*A-*trn*H region was amplified and sequenced by the primer pairs *psb*AF and *trn*HR [28]. The *atp*I-*atp*H region was amplified and sequenced using the primer pairs *atp*I and *atp*H [29]. The *trn*S-*trnf*M region was amplified and sequenced using the primer pair *trn*S-*trnf*M [30]. Table 3 contains the detail information.

**Table 3:** Primers used in this study.

Primer	Sequence(5′→3′)	Origin
ITS A	GGAAGGAGAAGTCGTAACAAGG	Mike *et al.* [26]
ITS B	CTTTTCCTCCGCTTATTGATATG	Mike *et al.* [26]
*trn*L-C	CGAAATCGGTAGACGCTACG	Taberlet *et al.* [27]
*trn*L-F	ATITGAACTGGTGACACGAG	Taberlet *et al.* [27]
*trn*L-MF	TAAAGAGAGAGTCCCATTTTAC	Liu *et al.* [11]
*trn*L-MR	GAGCGAGGAAGTAAAATGGGC	Liu *et al.* [11]
*trn*K-2R	AACTAGTCGGATGGAGTAG	Mike *et al.* [26]
*mat*K-19F	CGTTCTCATATTGCACTATG	Mike *et al.* [26]
*mat*K-969R	CTTTTCCTTGATATCGAACAT	Liu *et al.* [11]
*mat*K-731F	AAGAAAAGATTCTTTTGGTTCC	Liu *et al.* [11]
*psb*AF	GTTATGCATGAACGTAATGCTC	Sang *et al.* [28]
*trn*HR	CGCGCATGGTGGATTCACAAATC	Sang *et al.* [28]
*atp*I	TATTTACAAGYGGTATTCAAGCT	Shaw *et al.* [29]
*atp*H	CCAAYCCAGCAGCAATAAC	Shaw *et al.* [29]
*trn*S	GAGAGAGAGGGATTCGAACC	Demesure [30]
*trnf*M	CATAACCTTGAGGTCACGGG	Demesure [30]

The PCR products were run on 1.5% agarose gels to assess the quality of the amplified DNA. The gels with the target products were excised, purified using DNA Gel Extraction Kits (Axygen Biosciences), and then sequenced by Life Technologies Corporation.

### Sequence Editing and Assembling

Both forward and reverse sequences, as well as electropherograms were edited and assembled using DNASTAR (http://www.dnastar.com/). DNA sequences were aligned to the muscle model and manually adjusted using MEGA5.05 [31]. Aligned sequences are available from the corresponding authors upon request.

### Data Analyses

The datasets included a nuclear ITS, plastid DNA (cpDNA; including the *trn*L-F, *mat*K, *psb*A-*trn*H, *atp*I-*atp*H, *trn*S-*trnf*M), and their combination. Insertions, deletions, and some unavailable sequences were treated as missing. Phylogenetic analyses were performed under ML, MP, and Bayesian inference (BI) methods. The best-fit model for each dataset was selected by Modeltest 3.7 [32] under the Akaike Information Criterion (Table 2). The homogeneities between nrDNA ITS data and the combined cpDNA dataset were tested using the incongruence length difference (ILD) test [33], as implemented in PAUP* version 4.0b10 [34]. The ILD test was conducted with 1000 replicates, each with 10 random addition sequence replicates, TBR branch swapping, and keeping no more than 100 trees per random addition replicate. Following Cunningham [35], a significance level of P = 0.01 was adopted for this test.

MP analyses were performed using the PAUP* version 4.0b10 [34]. All characters were equally weighed and unordered. Test settings included 1000 replications of random addition sequence and heuristic search with tree bisection-reconnection branch swapping. Table 1 lists the tree length, consistency index (CI), and retention index (RI) ML analysis was performed using RAxML version 7.2.8 with 100 bootstrap replicates and settings as described in Stamatakis et al. [36]. BI analysis was performed using MrBayes 3.1.2 [37]. The best-fit model for each dataset was selected using Modeltest 3.7. In the combined dataset of all datasets, the model was also based on the best fit model for each individual dataset. The following settings were applied: sampling frequency = 1000, temp = 0.1, burn-in = 10 000, and number of Markov chain Monte Carlo generations = 40 000 000. The first 10 000 trees were discarded as burn-in to ensure that the chains reached stationarity. A majority-rule consensus tree was constructed on these trees sampled after generation 10 000 000.

Phylogenetic analyses were performed under the ML, MP, and BI for each dataset. The BI, MP, and ML trees of each dataset had similar topological structures, indicating the very good repeatability of the analysis and reliability of the experimental data. The differences among the BI, MP, and ML trees were the values of bootstrap percentages or posterior probabilities in each node. Generally, the values in the BI tree were higher than those in the MP and ML trees (Figs.1, S1, S2, S3, S4, S5, S6). We submitted all data and trees to TreeBase, http://purl.org/phylo/treebase/phylows/study/TB2:S13699.

### Nomenclature Acts

The electronic version of this article in Portable Document Format (PDF) in a work with an ISSN or ISBN will represent a published work according to the International Code of Nomenclature for algae, fungi, and plants, and hence the new names contained in the electronic publication of a PLOS ONE article are effectively published under that Code from the electronic edition alone, so there is no longer any need to provide printed copies.

In addition, new names contained in this work have been submitted to IPNI, from where they will be made available to the Global Names Index. The IPNI LSIDs can be resolved and the associated information viewed through any standard web browser by appending the LSID contained in this publication to the prefix http://ipni.org/. The online version of this work is archived and available from the following digital repositories: PubMed Central, LOCKSS.

## Supporting Information

Figure S1 **Bayesian consensus trees based on ITS data.** The Bayesian posterior probability (×100) is given above the branches.
(TIF)

Figure S2 **Maximum likelihood (ML) trees of ITS computed by RAxML with 100 bootstrap replicates.** The bootstrap values are given above the branches.
(TIF)

Figure S3 **Strict consensus tree of most parsimonious trees based on ITS data.** Tree length = 991 steps, CI = 0.5550, and RI = 0.7442. The bootstrap values of the maximum parsimony analysis are given above the branches.
(TIF)

Figure S4 **Bayesian consensus trees based on cpDNA combined dataset.** The Bayesian posterior probability (×100) is given above the branches.
(TIF)

Figure S5 **Maximum likelihood (ML) trees of cpDNA combined dataset computed by RAxML with 100 bootstrap replicates.** The bootstrap values are given above the branches.
(TIF)

Figure S6 **Strict consensus tree of most parsimonious trees based on cpDNA combined dataset.** Tree length = 1885 steps, CI = 0.7220, and RI = 0.7720. The bootstrap values of the maximum parsimony analysis are given above the branches.
(TIF)

Figure S7 ***Pendulorchis gaoligongensis*** **G. Q. Zhang, Ke Wei Liu et Z. J. Liu.** a. Plant on tree trunk, b. Flowering plant; c. Inflorescence; d. Flower, front view; e. Pollinarium, front view; f. Pollinarium, back view; g. Flower, side view.
(TIF)

Figure S8 ***Holcoglossum singchianum*** **G. Q. Zhang, L. J. Chen et Z. J. Liu.** a. Flowering in cultivation; b. Inflorescence; c and d. Flower, front view and side view; e and f. Pollinarium, front and back views.
(TIF)

Table S1 **Species and gene regions sequenced for analysis, as well as GenBank accession numbers.**
(DOC)
