# Supplementary figures and images for: A New Molecular Phylogeny and a New Genus, Pendulorchis, of the Aerides–Vanda Alliance (Orchidaceae: Epidendroideae)
Source: PLoS One. 2013 Apr 5;8(4):e60097. doi: 10.1371/journal.pone.0060097 (PMC3618120; doi:10.1371/journal.pone.0060097)

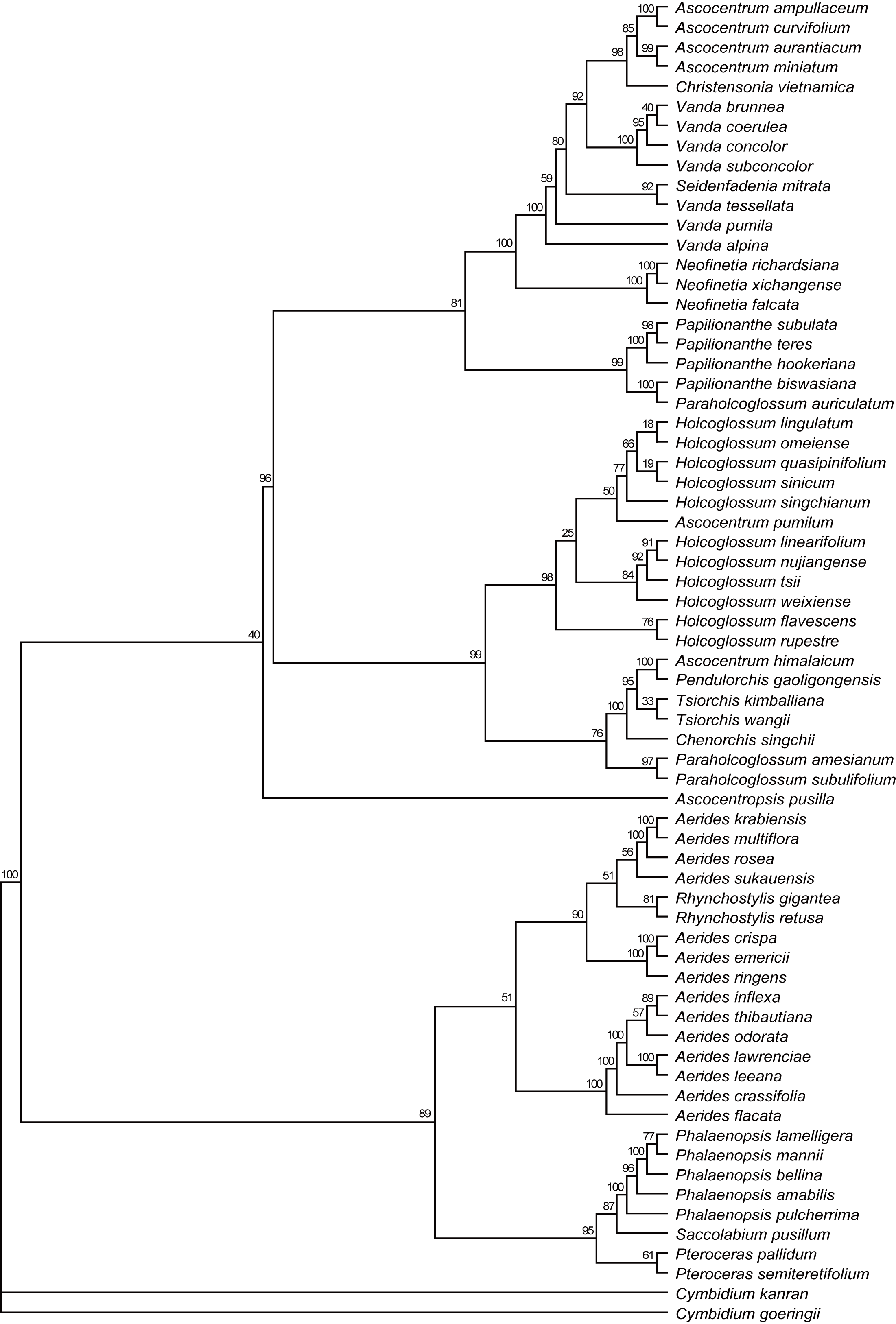

Supplement: Figure S1 — Bayesian consensus trees based on ITS data. The Bayesian posterior probability (×100) is given above the branches. (TIF) [file pone.0060097.s001.tif]

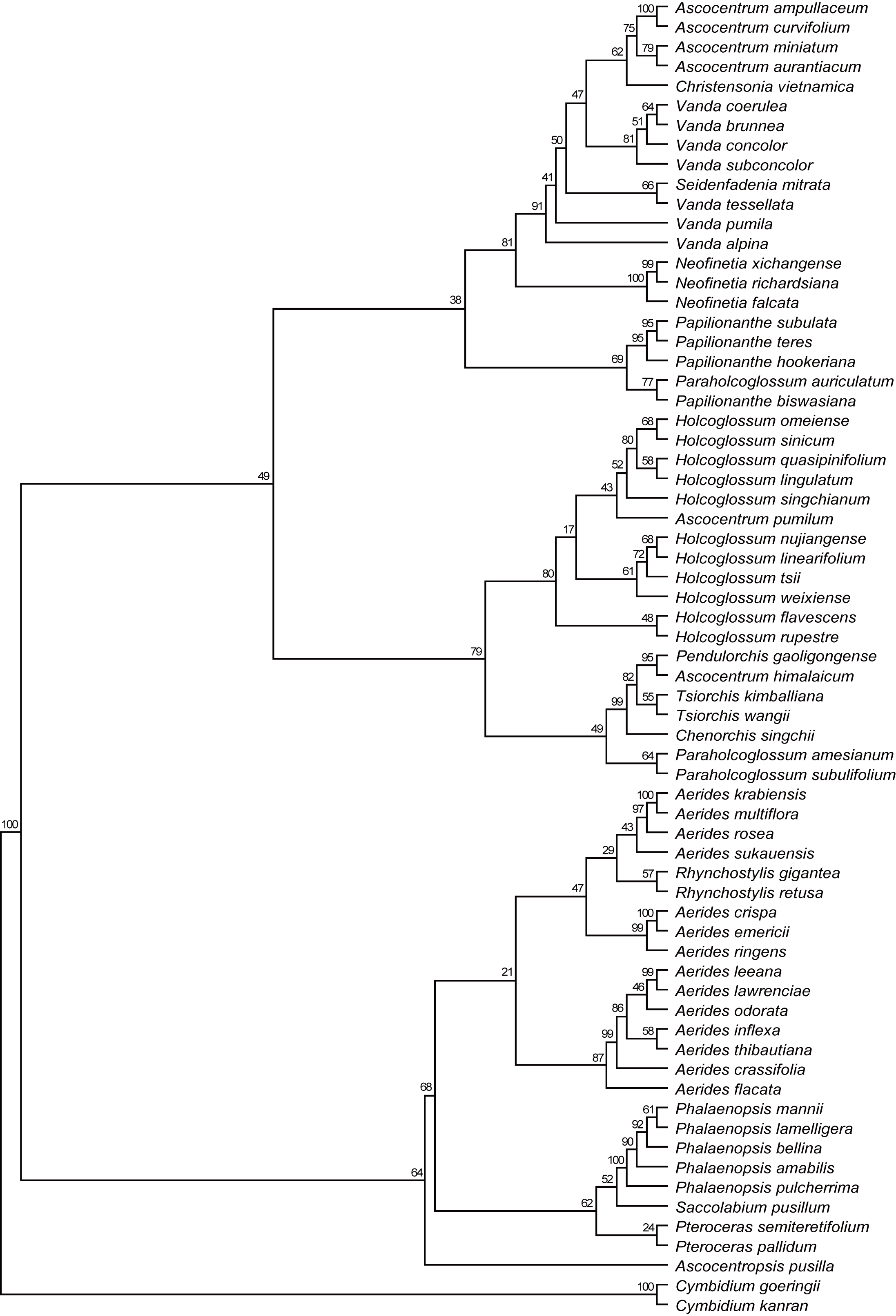

Supplement: Figure S2 — Maximum likelihood (ML) trees of ITS computed by RAxML with 100 bootstrap replicates. The bootstrap values are given above the branches. (TIF) [file pone.0060097.s002.tif]

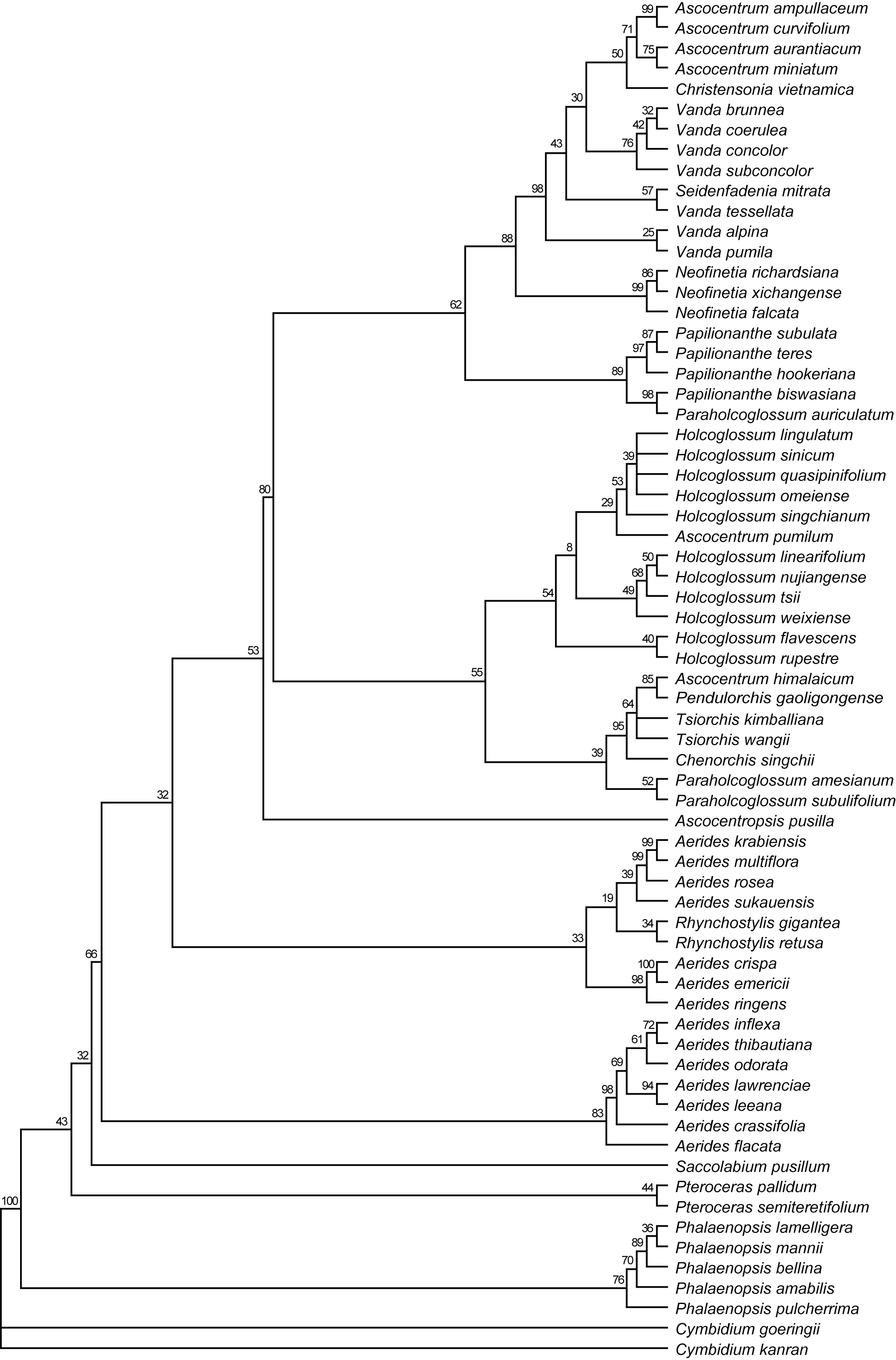

Supplement: Figure S3 — Strict consensus tree of most parsimonious trees based on ITS data. Tree length = 991 steps, CI = 0.5550, and RI = 0.7442. The bootstrap values of the maximum parsimony analysis are given above the branches. (TIF) [file pone.0060097.s003.tif]

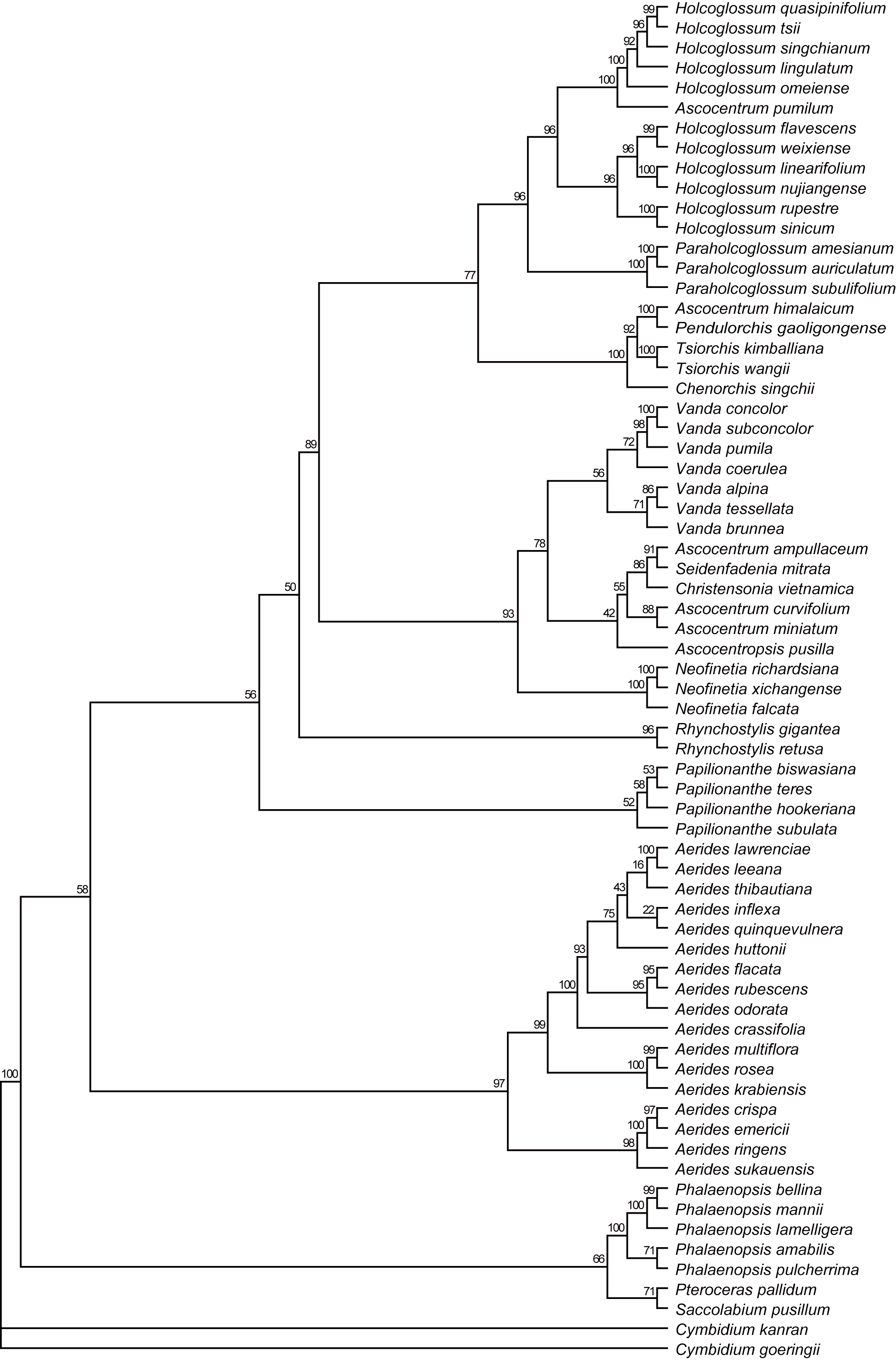

Supplement: Figure S4 — Bayesian consensus trees based on cpDNA combined dataset. The Bayesian posterior probability (×100) is given above the branches. (TIF) [file pone.0060097.s004.tif]

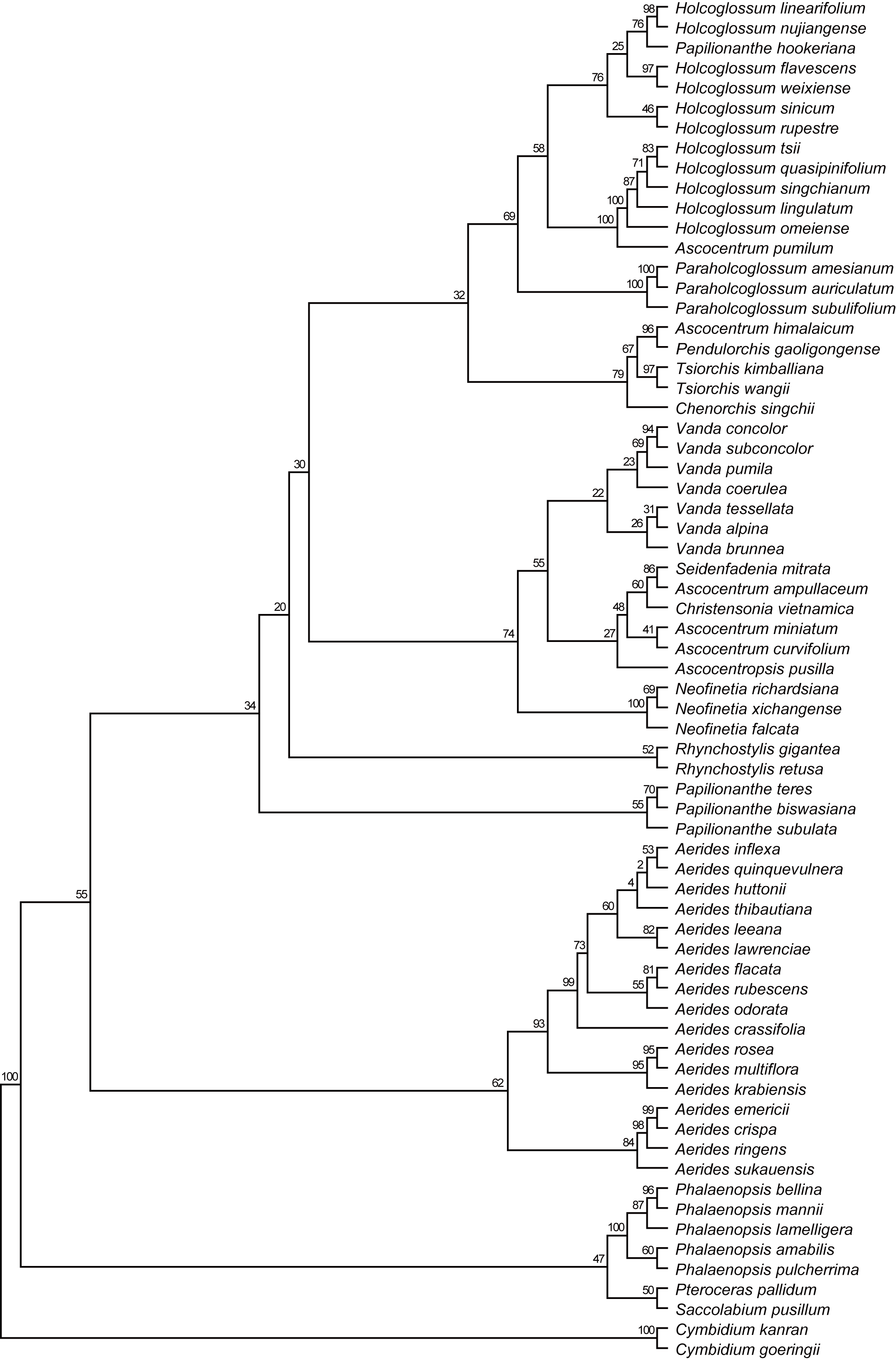

Supplement: Figure S5 — Maximum likelihood (ML) trees of cpDNA combined dataset computed by RAxML with 100 bootstrap replicates. The bootstrap values are given above the branches. (TIF) [file pone.0060097.s005.tif]

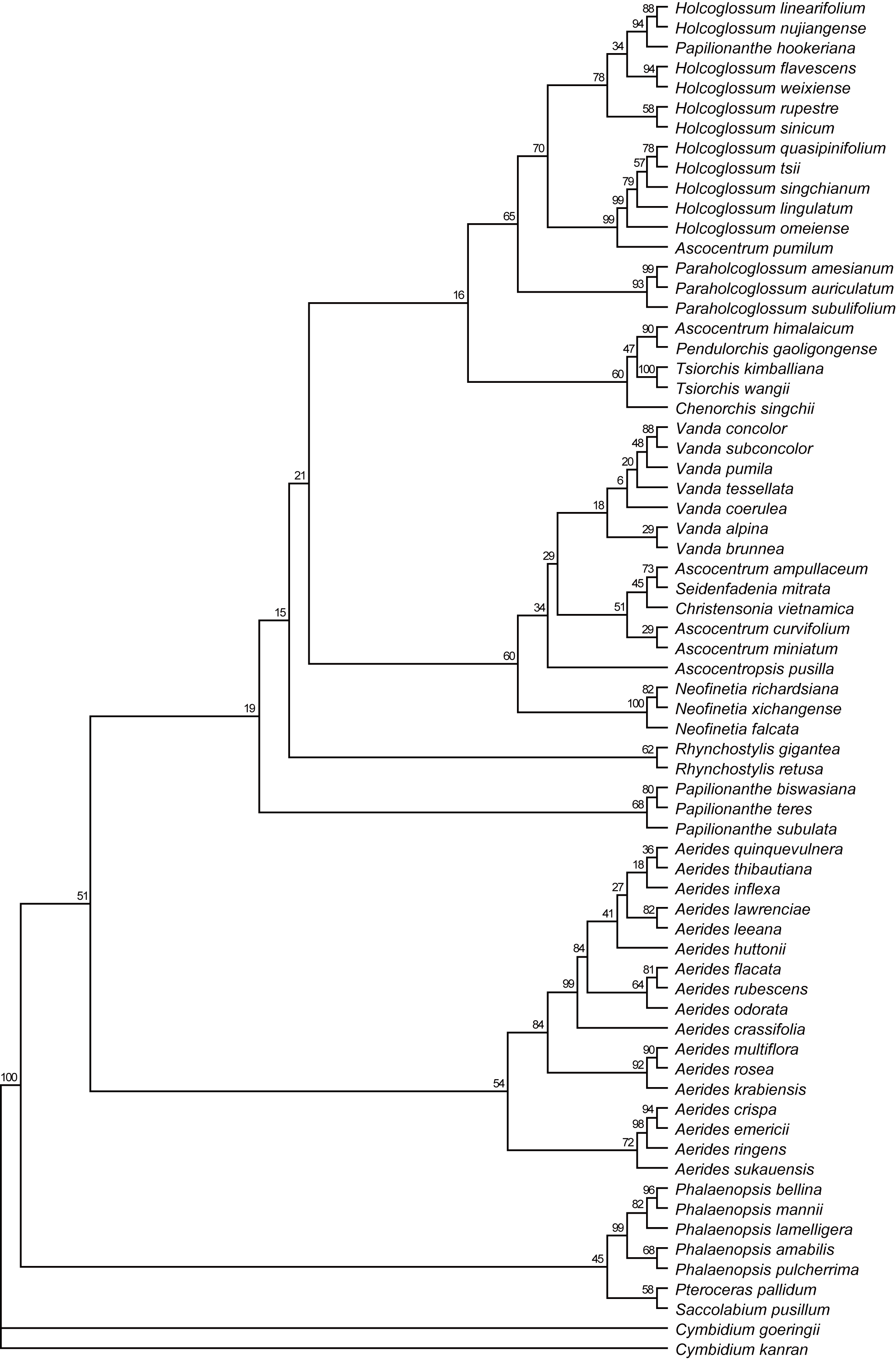

Supplement: Figure S6 — Strict consensus tree of most parsimonious trees based on cpDNA combined dataset. Tree length = 1885 steps, CI = 0.7220, and RI = 0.7720. The bootstrap values of the maximum parsimony analysis are given above the branches. (TIF) [file pone.0060097.s006.tif]

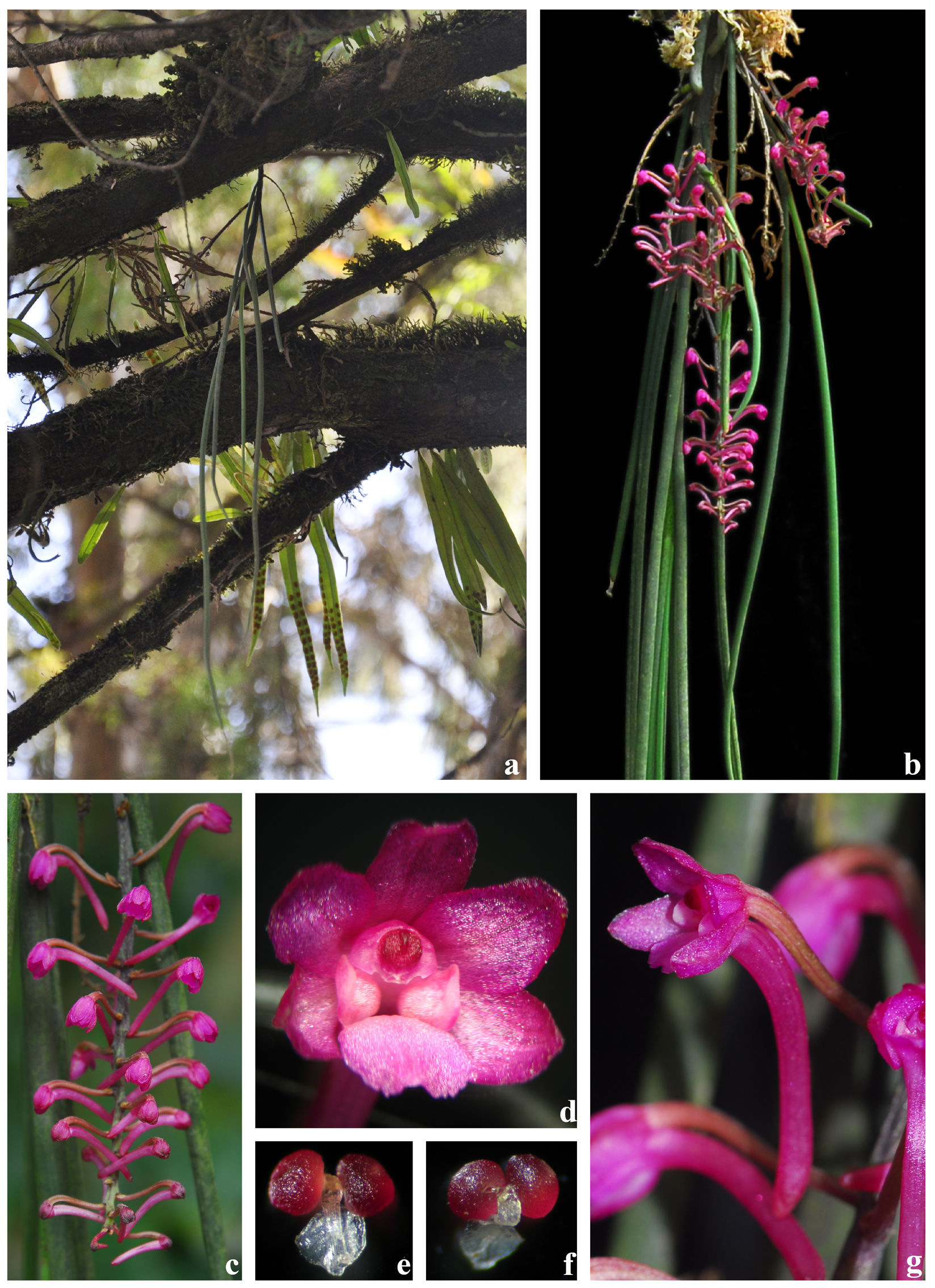

Supplement: Figure S7 — Pendulorchis gaoligongensis G. Q. Zhang, Ke Wei Liu et Z. J. Liu. a. Plant on tree trunk, b. Flowering plant; c. Inflorescence; d. Flower, front view; e. Pollinarium, front view; f. Pollinarium, back view; g. Flower, side view. (TIF) [file pone.0060097.s007.tif]

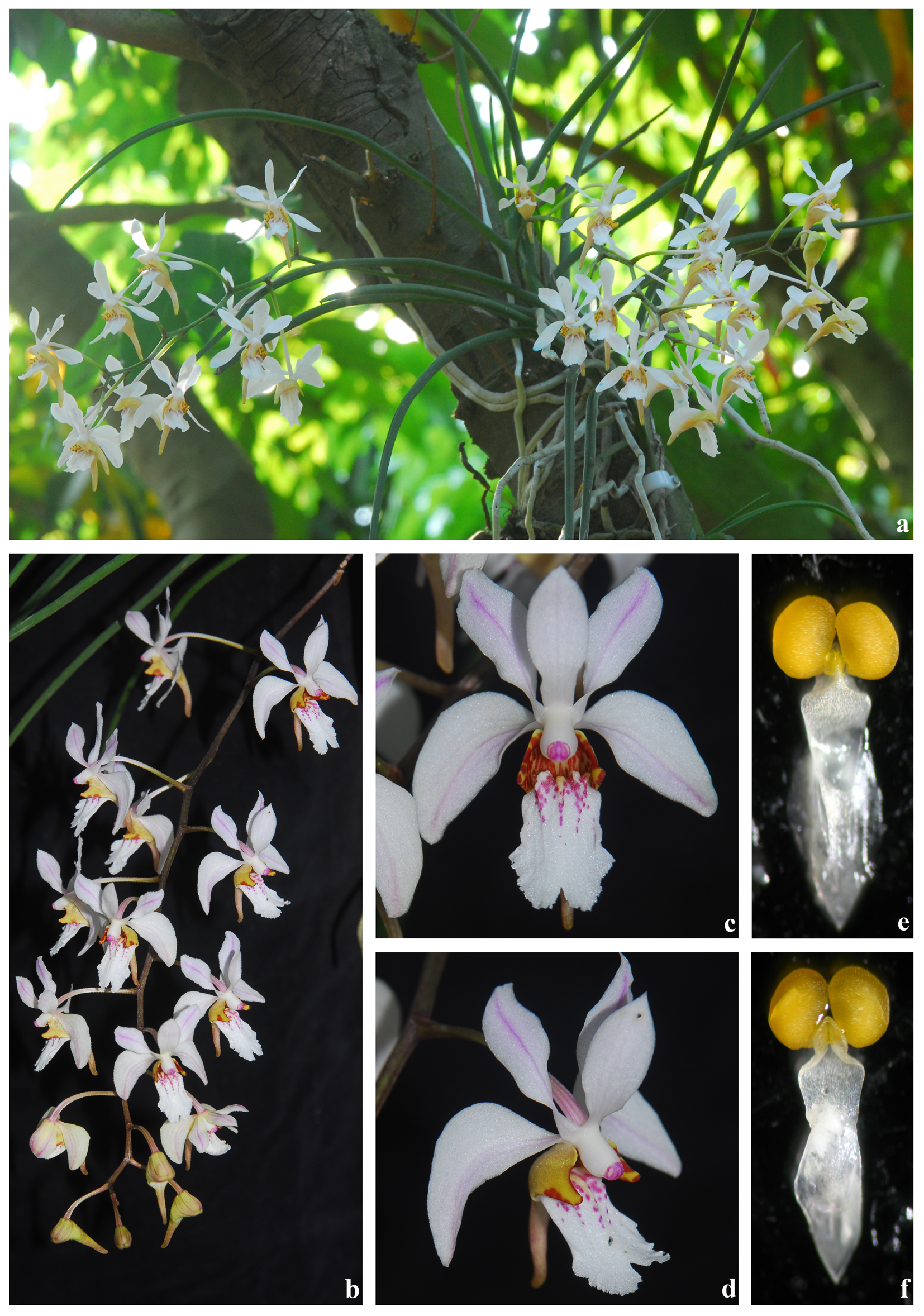

Supplement: Figure S8 — Holcoglossum singchianum G. Q. Zhang, L. J. Chen et Z. J. Liu. a. Flowering in cultivation; b. Inflorescence; c and d. Flower, front view and side view; e and f. Pollinarium, front and back views. (TIF) [file pone.0060097.s008.tif]
