# Supplementary material for: A New Molecular Phylogeny and a New Genus, Pendulorchis, of the Aerides–Vanda Alliance (Orchidaceae: Epidendroideae)
Source: PLoS One. 2013 Apr 5;8(4):e60097. doi: 10.1371/journal.pone.0060097 (PMC3618120; doi:10.1371/journal.pone.0060097)
Supplement: Table S1 — Species and gene regions sequenced for analysis, as well as GenBank accession numbers. (DOC) [file pone.0060097.s009.doc]

**Table S1.** Species and gene regions sequenced for analysis, as well as GenBank accession numbers.

| **Species** | **Voucher**  **(NOCC)** | **ITS** | ***mat*K** | ***trn*L-F** | ***psb*A*-trn*H** | ***atp*I*-atp*H** | ***trnS-trnf*M** |
| --- | --- | --- | --- | --- | --- | --- | --- |
| *Aerides crassifolia* |  | EF670350 | EF655785 | EF670402 | – | – | – |
| *A*. *crispa* |  | EF670351 | EF655793 | EF670407 | – | – | – |
| *A*. *emericii* |  | EF670320 | EF655792 | – | – | – | – |
| *A*. *flacata* | *Z. J. Liu 4973* | JX202646 | JX202664 | JX202716 | JX202681 | JX202611 | JX202734 |
| *A*. *huttonii* |  | – | EF655816 | EF670400 | – | – | – |
| *A*. *inflexa* |  | EF670322 | EF655775 | EF670391 | – | – | – |
| *A*. *krabiensis* |  | EF670341 | EF655784 | EF670404 | – | – | – |
| *A*. *lawrenciae* |  | EF670328 | EF655781 | EF670393 | – | – | – |
| *A*. *leeana* |  | EF670327 | EF655797 | EF670396 | – | – | – |
| *A*. *multiflora* |  | AY912258 | EF655773 | EF670403 | – | – | – |
| *A*. *odorata* |  | AB217529 | EF655815 | EF670389 | – | – | – |
| *A*. *quinquevulnera* |  | –- | EF655808 | EF670394 | – | – | – |
| *A*. *ringens* |  | EF670353 | EF655791 | EF670406 | – | – | – |
| *A*. *rosea* | *Z. J. Liu 3477* | EF670340 | EF655787 | EF670405 | JX202682 | JX202612 | JX202735 |
| *A*. *rubescens* |  | – | EF655779 | EF670392 | – | – | – |
| *A*. *sukauensis* |  | EF670355 | EF655779 | EF670408 | – | – | – |
| *A*. *thibautiana* |  | EF670337 | EF655813 | EF670398 | – | – | – |
| *Ascocentropsis pusilla* |  | DQ091677 | EF079273 | – | – | – | – |
| *Ascocentrum ampullaceum* | *Z. J. Liu 4789* | AY912260 | EF655808 | JX202718 | JX202683 | JX202613 | JX202736 |
| *A*. *aurantiacum* |  | DQ091677 | – | – | – | – | – |
| *A*. *curvifolium* | *Z. J. Liu 3348* | JX202648 | JX202666 | JX202719 | JX202684 | JX202614 | JX202737 |
| *A*. *himalaicum* | *Z. J. Liu 5127* | JX202649 | JX202667 | JX202720 | JX202685 | JX202615 | JX202738 |
| *A*. *lushuiense* | *Z. J. Liu 5082* | JX202650 | JX202668 | JX202721 | JX202686 | JX202616 | JX202739 |
| *A*. *miniatum* |  | DQ091678 | – | – | – | – | – |
| *A*. *pumilum* | *Z. J. Liu 6240* | JX202651 | JX202669 | JX202722 | JX202687 | JX202617 | JX202740 |
| *Chenorchis singchii* | *Z. J. Liu 3879* | JX202652 | JX202670 | JX202623 | JX202688 | JX202618 | JX202741 |
| *Christensonia vietnamica* |  | EF670357 | EF079272 | EF670413 | – | – | – |
| *Cymbidium goeringii* | *Z. J. Liu 2522* | JX202653 | JX202671 | JX202624 | JX202689 | JX202619 | JX202742 |
| *C*. *kanran* | *Z. J. Liu 3184* | JX202654 | JX202672 | JX202725 | JX202690 | JX202620 | JX202743 |
| *Holcoglossum flavescens* | *Z. J. Liu 2659* | HQ452904 | HQ452919 | HQ452934 | JX202691 | JX202621 | JX202744 |
| *H*. *linearifolium* | *Z. J. Liu 4865* | JN106337 | JN106351 | JN106344 | JX202692 | JX202622 | JX202745 |
| *H*. *lingulatum* | *Z. J. Liu 3544* | JN106334 | JN106348 | JN106341 | JX202693 | JX202623 | JX202746 |
| *H*. *nujiangense* | *Z. J. Liu 3498* | HQ452908 | HQ452923 | HQ452938 | JX202694 | JX202624 | JX202747 |
| *H*. *omeiense* | *Z. J. Liu 2652* | JN106332 | JN106346 | JN106339 | JX202695 | JX202625 | JX202748 |
| *H*. *quasipinifolium* | *Z. J. Liu 2974* | HQ452909 | HQ452924 | HQ452939 | JX202696 | JX202626 | JX202749 |
| *H*. *rupestre* | *Z. J. Liu 2658* | HQ452905 | ER558948 | HQ452935 | JX202697 | JX202627 | JX202750 |
| *H*. *singchianum* | *Z. J. Liu 4532* | JX202655 | JX202673 | JX202726 | JX202698 | JX202628 | JX202751 |
| *H*. *sinicum* | *Z. J. Liu 2664* | HQ452906 | EU558956 | HQ452936 | JX202699 | JX202629 | JX202752 |
| *H*. *tsii* | *Z. J. Liu 3656* | EU558927 | AB217732 | EU558902 | JX202700 | JX202630 | JX202753 |
| *H*. *weixiense* | *Z. J. Liu 2663* | HQ452900 | EU558957 | HQ452930 | JX202701 | JX202631 | JX202754 |
| *Neofinetia falcata* | *Z. J. Liu 4813* | JX202656 | JX202674 | JX202727 | JX202702 | JX202632 | JX202755 |
| *N*. *richardsiana* | *Z. J. Liu 4812* | JX202657 | JX202675 | JX202728 | JX202703 | JX202633 | JX202756 |
| *N*. *xichangense* | *Z. J. Liu 4966* | JX202658 | JX202676 | JX202729 | JX202704 | JX202634 | JX202757 |
| *Papilionanthe biswasiana* | *Z. J. Liu 4815* | HQ452914 | HQ452929 | HQ452944 | JX202705 | JX202635 | JX202758 |
| *P*. *hookeriana* |  | FJ361770 | FJ495167 | – | – | – | – |
| *P*. *subulata* |  | AB217568 | AB217744 | – | – | – | – |
| *P*. *teres* | *Z. J. Liu 3721* | EU558934 | EU558937 | EU558872 | JX202706 | JX202636 | JX202659 |
| *Paraholcoglossum amesianum* | *Z. J. Liu 2716* | JN106336 | JN106350 | JN106343 | JX202707 | JX202637 | JX202660 |
| *P*. *auriculatum* | *Z. J. Liu 2758* | HQ452913 | HQ452928 | HQ452943 | JX202708 | JX202638 | JX202661 |
| *P*. *subulifolium* | *Z. J. Liu 3249* | JN106335 | JN106349 | JN106342 | JX202709 | JX202639 | JX202662 |
| *Phalaenopsis amabilis* |  | AY391523 | AB217747 | AY273653 | FJ460397 | – | – |
| *P*. *bellina* |  | AF537015 | EU251961 | AY273632 | – | – | – |
| *P*. *lamelligera* |  | AY912233 | EU179845 | AY273679 | – | – | – |
| *P*. *mannii* |  | AY228496 | AY121744 | AF519969 | – | – | – |
| *P*. *pulcherrima* |  | AF536993 | EF079282 | AY273659 | – | – | – |
| *Pteroceras pallidum* |  | AB217578 | AB217754 | – | – | – | – |
| *P*. *semiteretifolium* |  | DQ091722 | – | – | – | – | – |
| *Rhynchostylis gigantea* |  | GQ251304 | EF655778 | EF670411 | GQ251330 | – | – |
| *R*. *retusa* |  | EU558933 | EU558938 | EU558873 | GQ251334 | – | – |
| *Saccolabium pusillum* |  | AB217580 | AB217756 | – | – | – | – |
| *Seidenfadenia mitrata* |  | EF670380 | AB217762 | EF670414 | – | – | – |
| *Tsiorchis kimballiana* | *Z. J. Liu 2114* | JN106331 | JN106345 | JN106338 | JX202710 | JX202640 | JX202763 |
| *T*. *wangii* | *Z. J. Liu 2818* | JN106333 | JN106347 | JN106340 | JX202711 | JX202641 | JX202764 |
| *Vanda alpina* |  | – | – | GU185933 | – | – | – |
| *V*. *brunnea* | *Z. J. Liu 2422* | JX202660 | JX202677 | JX202730 | JX202712 | JX202642 | JX202765 |
| *V*. *coerulea* | *Z. J. Liu 2937* | JX202661 | JX202678 | JX202731 | JX202713 | JX202643 | JX202766 |
| *V*. *concolor* | *Z. J. Liu 3335* | JX202662 | JX202679 | JX202732 | JX202714 | JX202644 | JX202767 |
| *V*. *pumila* |  | EF670372 | AB217770 | EU558876 | – | – | – |
| *V*. *subconcolor* | *Z. J. Liu 3333* | JX202663 | JX202680 | JX202733 | JX202715 | JX202645 | JX202768 |
| *V*. *tessellata* |  | DQ091679 | JN004626 | GU185939 | – | – | – |
